# Prevalence and distribution of soil-borne zoonotic pathogens in Lahore district of Pakistan

**DOI:** 10.3389/fmicb.2015.00917

**Published:** 2015-09-10

**Authors:** Muhammad Z. Shabbir, Tariq Jamil, Asad A. Ali, Arfan Ahmad, Muhammad Naeem, Muhammad H. Chaudhary, Muhammad Bilal, Muhammad A. Ali, Khushi Muhammad, Tahir Yaqub, Asghari Bano, Ali I. Mirza, Muhammad A. B. Shabbir, Walter R. McVey, Ketan Patel, Stephen Francesconi, Bhushan M. Jayarao, Masood Rabbani

**Affiliations:** ^1^University of Veterinary and Animal SciencesLahore, Pakistan; ^2^Quaid-i-Azam UniversityIslamabad, Pakistan; ^3^University of the PunjabLahore, Pakistan; ^4^Government College UniversityLahore, Pakistan; ^5^Department of Veterinary and Biomedical Sciences, The Pennsylvania State UniversityUniversity Park, PA, USA; ^6^Naval Medical Research UnitFrederick, MA, USA

**Keywords:** *Bacillus anthracis*, *Francisella tularensis*, *Coxiella burnetii*, *Burkholderia mallei/pseudomallei*, *Yersinia pestis*, soil chemistry, risk factors

## Abstract

A multidisciplinary, collaborative project was conducted to determine the prevalence and distribution of soil-borne zoonotic pathogens in Lahore district of Pakistan and ascertain its Public Health Significance. Using a grid-based sampling strategy, soil samples (*n* = 145) were collected from villages (*n* = 29, 5 samples/village) and examined for *Bacillus anthracis, Burkholderia mallei/pseudomallei, Coxiella burnetii, Francisella tularensis*, and *Yersinia pestis* using real time PCR assays. Chemical analysis of soil samples was also performed on these samples. The relationship between soil composition and absence or presence of the pathogen, and seven risk factors was evaluated. DNA of *B. anthracis* (CapB), *B. mallei/pseudomallei* (chromosomal gene)*, C. burnetii* (IS1111, transposase gene), and *F. tularensis* (lipoprotein/outer membrane protein) was detected in 9.6, 1.4, 4.8, and 13.1% of soil samples, respectively. None of the samples were positive for protective antigen plasmid (PA) of *B. anthracis* and *Y. pestis* (plasminogen activating factor, pPla gene). The prevalence of *B. anthracis* (CapB) was found to be associated with organic matter, magnesium (Mg), copper (Cu), chromium (Cr), manganese (Mn), cobalt (Co), cadmium (Cd), sodium (Na), ferrous (Fe), calcium (Ca), and potassium (K). Phosphorous (P) was found to be associated with prevalence of *F. tularensis* while it were Mg, Co, Na, Fe, Ca, and K for *C. burnetii*. The odds of detecting DNA of *F. tularensis* were 2.7, 4.1, and 2.7 higher when soil sample sites were >1 km from animal markets, >500 m from vehicular traffic roads and animal density of < 1000 animals, respectively. While the odds of detecting DNA of *C. burnetii* was 32, 11.8, and 5.9 higher when soil sample sites were >500 m from vehicular traffic roads, presence of ground cover and animal density of < 1000 animals, respectively. In conclusion, the distribution pattern of the soil-borne pathogens in and around the areas of Lahore district puts both human and animal populations at a high risk of exposure. Further studies are needed to explore the genetic nature and molecular diversity of prevailing pathogens together with their seroconversion in animals and humans.

## Introduction

Pathogens of public health significance such as *Burkholderia mallei/pseudomallei, Francisella tularensis, Yersinia pestis, Bacillus anthracis*, and *Coxiella burnetii* have significantly influenced human health throughout history (Sjöstedt, 2007; Ayyadurai et al., 2008; Oyston, 2008; Butler, 2009; Khan et al., 2013a) and anticipated to do so for the foreseeable future. These pathogens may enter the human body either through ingestion, inhalation or contact with contaminated soil and infected animals (Coenye and Vandamme, 2003; Ayyadurai et al., 2008; Oyston, 2008; Kersh et al., 2013; Khan et al., 2013a).

The incidence of *B. anthracis, F. tularensis, B. mallei/pseudomallei* and *Y. pestis* have been reported worldwide (Ayyadurai et al., 2008; Oyston, 2008; Butler, 2009). Prevalence of *B. anthracis* and *B. mallei* in humans and animals has been previously reported in Punjab province of Pakistan based on clinical findings and not supported by laboratory based confirmation. Further, little is known about the epidemiology of *F. tularensis* and *Y. pestis* in human and animal populations in Pakistan. Determination of the true prevalence of these pathogens is further complicated due to lack of a reporting system in the province of Punjab. The objectives of this study were to; (1) determine the prevalence of soil-borne zoonotic pathogens including *B*. *anthracis, B. mallei/pseudomallei, F. tularensis, Y. pestis* and *C. burnetii* in soil samples collected from villages in Lahore district, (2) identify soil components that favor or deter the presence of pathogens, and (3) identify risk factors associated with the presence or absence of the pathogen in the soil. It is anticipated that the findings of the study will provide information to undertake rigorous epidemiologic investigations focused on prevalence and distribution of soil-borne zoonotic pathogens in humans and domestic animals in Pakistan.

## Materials and methods

### Study area

**Figure 1:**
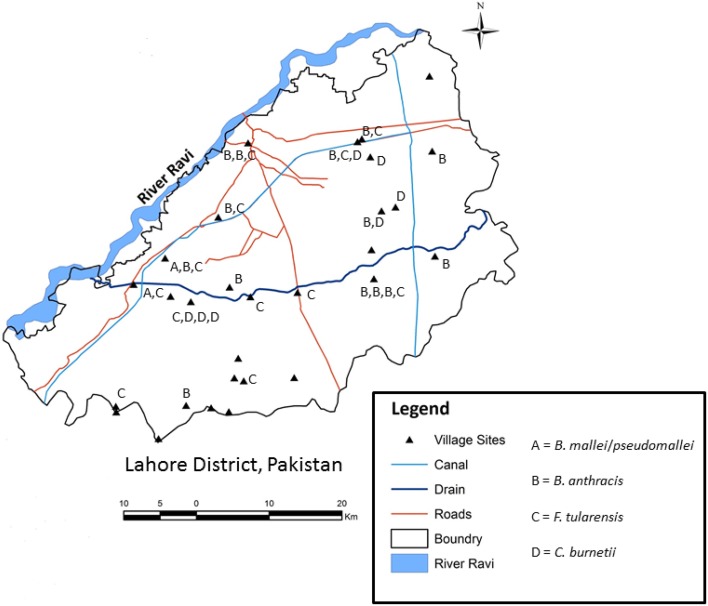
**Geospatial distribution of ***Burkholderia mallei***/***pseudomallei***, ***Bacillus anthracis***, ***Francisella tularensis***, and ***Coxiella burnetii*** DNA in soil samples collected in Lahore district, Pakistan**.

The Lahore district in Punjab Province of Pakistan was selected for conducting the study. Lahore (31°15′–31°45′N and 74°01′–74°39′E) is bound in the north and west by the Sheikhupura district, Kasur district in the south and India in the east. The river Ravi flows on the north-western side of Lahore city (Figure 1). The study area is 217 m above sea level and covers a total land area of 1772 km^2^. With a population exceeding 10 million, administratively the district is comprised of 10 towns, 271 union councils and 301 villages. Villages represent the lowest administrative unit, while a collection of villages represent a union council.

### Sample collection

Lahore district has 301 villages. Ten percent of these villages (*n* = 29) were randomly selected using Open Epi version 2.3.1 (http://www.openepi.com/Menu/OE_Menu.htm) software using a 5% confidence limit and 80% level of significance (Figure 1). From each village, five sites were identified and sampled by removing 3–5 inches of the top soil. Samples from four sites were locations that had both animals and humans dwelling in close proximity while the fifth site was located in an area where there was no evidence of apparent recent human and animal activity. Samples were collected using personal protective equipment (PPE). From each sample site, three separate samples were collected approximately within one square meter of the sample site. The three samples were pooled, mixed thoroughly and divided into three aliquots for soil chemistry (~500 g), real time (RT) PCR assays for pathogens (~200 g) and sample archives (~500 g). Using a sample collection survey questionnaire, necessary information pertaining to risk factor analysis for a given sample collection site including GIS location, presence or absence of domestic animals, animal density, distance from animal market, main road and water source such as river, canal, streams, and drains (canal or channel system which carries sewage and rain water), presence or absence of vegetation and human dwelling was obtained.

### DNA extraction from soil samples

DNA from soil samples was extracted using PowerSoil® DNA Isolation Kit (MoBio, USA) as per the manufacturer's instructions. The quality (A_260/280_ and A_260/230_) of the DNA was assessed by spectrophotometry (NanoDrop, USA) while the quantity (ng/μL) was determined by Qubit fluorometer (Invitrogen, USA) using the DNA BR Assay kit (Invitrogen, USA) as per the manufacturer's instruction.

### Real time PCR analysis

The prevalence of selected soil-borne pathogens was determined by RT PCR (CFX96, BioRad, USA). Previously reported oligonucleotides (primers and Taqman probes) were used for detection of *B. anthracis*, [plasmids containing capsular (CapB) and protective antigen (PA)], *Y. pestis* (Plasminogen activating factor, pPla gene), *F. tularensis* (Outer membrane) (Christensen et al., 2006), *B. mallei/pseudomallei* (Chromosomal gene) (this study) and *C. burnetii* (IS1111 gene) (Tozer et al., 2014) (Table 1).

**Table 1:** **List of PCR primers and probes used in this study**.

**Pathogen**	**Gene^a^^,^^b^^,^^c^**		**Sequence (5′–3′)**
*Bacillus anthracis*	Capsular antigen (pXO2)^a^	FP	CAGATAATGCATCGCTTGCTTTAG
		RP	GGATGAGCATTCAACATACCACG
		Probe	CAGAGGCTCTTGGATTGATGAGGAAACAO
	Protective antigen (pXO1)^a^	FP	TTCAAGTTGTACTGGACCGATTCTC
		RP	TCCATCATTGTCACGGTCTGG
		Probe	CCGTAGGTCCAGCACTTGTACTTCGCTTO
*Yersinia pestis*	Plasminogen activating factor (pPla)^a^	FP	ATTGGACTTGCAGGCCAGTATC
		RP	ATAACGTGAGCCGGATGTCTTC
		Probe	AAATTCAGCGACTGGGTTCGGGCACA
*Burkholderia mallei/pseudomallei*	Chromosomal gene^b^	FP	GCAAATCACCTTCGATGCAAC
		RP	CTAATTGAACCGAACCTTCG
		Probe	TCATCGCGACACTGGAATCGATGCGACACAT
*Francisella tularensis*	Lipoprotein/outer membrane protein^a^	FP	CAGCATACAATAATAACCCACAAGG
		RP	TCAGCATACTTAGTAATTGGGAAGC
		Probe	TTACAATGGCAGGCTCCAGAAGGTT
*Coxiella burnetii*	IS1111 gene/Transposase^c^	FP	GTCTTAAGGTGGGCTGCGTG
		RP	CCCCGAATCTCATTGATCAGC
		Probe	AGCGAACCATTGGTATCGGACGTTTATGG

a *Primers (FP, forward primer; RP, reverse primer) and probes used in the study were as described by Christensen et al. (2006)*.

b *this study*.

c *Primers and probes used in the study were as described by Tozer et al. (2014)*.

RT PCR assay was optimized and validated using the positive controls (dsDNA PCR products) and proficiency testing samples provided by Drs. Francesconi and Patel Naval Medical Research Unit, Frederick, Maryland. RT PCR assays for detection of *B. anthracis, B. mallei/pseudomallei, F. tularensis* and *Y. pestis* were performed in 25 μL of reaction volume comprising of final concentration of 1X PCR buffer, 5 mM of MgCl_2_, 0.25 mg/mL of bovine serum albumin, 0.25 mM of dNTPs, 0.6 μM of each forward and reverse primer, 0.025 μM of probe and 0.5 U of *Taq* polymerase along with DNA extracted from soil (10–30 ng). The cycling condition was as follows; one cycle of 95°C for 5 min followed by 45 cycles each of denaturation at 94°C for 5 s and annealing at 60°C for 20 s; and then one cycle of cooling at 40°C for 1 min. For detection of *C. burnetii*, the 25 μL of reaction volume contained 12.5 μL of qPCR mix (Thermo Scientific, USA), 10 pM of each primer and probe and 10–30 ng of template DNA. The cycling condition was as follows: 15 min incubation at 95°C followed by 50 cycles of 95°C for 15 s and 60°C for 1 min. Diethylpyrocarbonate (DEPC)-H_2_O was used as a negative control in all the assays. DNA from the same extract underwent two independent RT PCR reaction for the studied pathogens. Soil samples that exhibited a positive RT PCR result were assayed a third time beginning from genome extraction and the PCR products (presence and size in bp) were assessed by agarose gel electrophoresis (3.0%) with the appropriate controls and a 50 bp ladder (Fermentas, Germany).

### Soil chemistry analysis

The physical and chemical properties of soil samples were analyzed using validated techniques published previously for pH (McKeague, 1978), moisture (McLean, 1982), texture (Robert and Frederick, 1995), total soluble salts (Magistad et al., 1995), phosphorous (Frank et al., 2012), copper, chromium, nickel, manganese, cobalt, lead, cadmium, iron, sodium, potassium, calcium, and magnesium (Soltanpour and Schwab, 1977), nitrogen (Fierer et al., 2001) and organic matter (Nelson and Sommers, 1982).

### Data analysis

A total of 145 samples from 29 villages in Lahore district were examined for the presence of five pathogens. Results of the RT PCR analysis, soil chemistry and seven risk factors for each sample was compiled in a single Microsoft Excel spreadsheet. The presence or absence of a pathogen in relation to soil chemistry and seven potential risk factors was determined through student t distribution (*T*-test) and odd ratio (OR), respectively. Soil chemistry values (average count) were compared using the Tukey-Kramer (equal variance) or Dunnett's T3 (unequal variance) procedures. These two procedures were used due to unequal sample sizes observed in the two categories of a given value. The Tukey-Kramer procedure performs all pair-wise comparisons, testing whether the means are significantly different. The Dunnett's T3 procedure performs all comparisons with a control category. *P* < 0.05 was considered significant.

ORs were used to evaluate risk factors associated with pathogen. Observations to the seven risk factors were grouped by their categorical response (e.g., *yes, no*) to estimate if an observation had an influence on the presence or absence of the pathogen. For a potential risk factor, OR > 1 was considered to be associated with the outcome (presence or absence of pathogen) while it was considered vice versa for OR < 1. Confidence interval (CI, 95%) was used to estimate precision of the OR where a large CI value was considered a low level of precision and small CI was considered with higher precision. Statistical analyses related to soil chemistry were performed with SPSS (version17.0; SPSS Inc., Chicago, IL) and STATA (version 12.0, College Station, Texas, USA) while ORs were calculated through two by two frequency table available with OpenEpi version 2.3.1.

## Results

### Prevalence of DNA of bacterial pathogens in soil samples collected in Lahore district

A total of 29 of 301 villages in Lahore district were examined for soil-borne pathogens including *B. anthracis, B. mallei/pseudomallei, C. burnetii, F. tularensis* and *Y. pestis*. None of the soil samples were positive for DNA of *Y. pestis*. Soil samples from 25 of 29 (86.2%) villages and 40 of 145 (27.6%) sample sites were positive for DNA of atleast one of the four pathogens. DNA of *B. anthracis* (CapB), *F. tularensis* and *C. burnetii* was detected in 11 (37.9%), 14 (48.3%) and 4 (13.8%) of 29 villages, respectively. None of the samples were positive for the DNA encoding for protective antigen (pOX1). *Burkholderia mallei/pseudomallei* was detected in two of 29 (6.9%) villages and two of 145 (1. 4%) soil samples. Both these villages (Medhipur and Nasir Gurj) shared the same major roadway and were adjacent to a water canal (Table 2).

**Table 2:** **Soil sample sites positive for DNA of four soil-borne pathogens**.

	**Village (sample site)**	***Bacillus anthracis***	***Burkholderia mallei/pseudomallei***	***Coxiella burnetii***	***Francisella tularensis***
1	Bagrian			1	
2	Batapur	1			1
3	Bhaseen				4
4	Gawala Colony				1
5	Hadiara	1			
6	Halloki	1			
7	Halokeey				2
8	Hanjarwal			2	
9	Heir	3			1
10	Hira Singh	1			1
11	Jallo Pind	1			
12	Jiya Bagga				1
13	Kahna				1
14	Kot Bagh Ali			3	
15	Lakhodere				2
16	Mandhiyala	1			
17	Maraka				1
18	Medhipur	1	1		1
19	Nasir Gurj		1		
20	Paajiyan	1			
21	Pangali	1			
22	Raiwind Pind				1
23	Sanat Nagar	2			1
24	Sultankey				1
25	Tehra Pind			1	
	Total (%)	14 (9.6%)	2 (1.4%)	7 (4.8%)	19 (13.1%)

### Soil chemistry

The pH of soil samples collected from different sites ranged from near neutral to alkaline (6.50–9.85). A total of 20 soil analytes were examined including moisture (0.50–42.24%)_,_soluble salts (0.20–50.21%), organic matter (0.05–7.25 mg/Kg), clay (0.0–15.0 mg/Kg), sand (72.0–99.0 mg/Kg), silt (0.00–115.0 mg/Kg), nitrogen (0.002–3.92 mg/Kg), phosphorus (1.00–146.67 mg/Kg), magnesium (0.12–82.24 mg/Kg), copper (0.026–3.08 mg/Kg), chromium (0.002–0.25 mg/Kg), nickel (0.002–0.38 mg/Kg), manganese (0.015–0.89 mg/Kg), cobalt (0.005–0.15 mg/Kg), lead (0.01–1.44 mg/Kg), cadmium (0.03–0.47 mg/Kg), sodium (0.06–150.78 mg/Kg), ferric (0.04–5.59 mg/Kg), calcium (0.09–103.52 mg/Kg), and potassium (0.12–194.84 mg/Kg). Among the analytes examined, the concentration of analytes including moisture, soluble salts, clay, sand, silt, phosphorus, magnesium, sodium, calcium, and potassium were particularly variable (Table 3).

**Table 3:** **Concentration of soil analytes from villages in Lahore district**.

**Soil analyte**	**Mean**	**Range**	**Standard deviation**	**Standard error**
pH	8.26	6.50–9.85	0.503	0.042
Moisture (%)	9.12	0.50–42.2	6.225	0.517
Soluble salts (%)	3.71	0.20–50.1	5.772	0.479
Organic matter	2.46	0.05–7.25	1.565	0.130
Clay (mg/Kg)	6.02	0.00–15.0	3.728	0.310
Sand (mg/Kg)	85.44	72.0–99.0	5.985	0.497
Slit (mg/Kg)	10.02	0.00–115	12.936	1.074
Nitrogen (mg/Kg)	0.18	0.002–3.92	0.371	0.031
Phosphorus (mg/Kg)	39.85	1.00–146.7	37.164	3.086
Magnesium (mg/Kg)	10.74	0.12–82.24	15.525	1.289
Copper (mg/Kg)	0.41	0.026–3.08	0.413	0.034
Chromium (mg/Kg)	0.13	0.002–0.25	0.060	0.005
Nickel (mg/Kg)	0.04	0.002–0.38	0.039	0.003
Manganese (mg/Kg)	0.07	0.015–0.89	0.127	0.011
Cobalt (mg/Kg)	0.07	0.005–0.15	0.046	0.004
Lead (mg/Kg)	0.44	0.01–1.44	0.224	0.019
Cadmium (mg/Kg)	0.22	0.03–0.47	0.112	0.009
Sodium (mg/Kg)	25.13	0.06–150.8	25.314	2.102
Ferric (mg/Kg)	0.59	0.04–5.59	0.904	0.075
Calcium (mg/Kg)	21.65	0.09–103.5	29.435	2.444
Potassium (mg/Kg)	34.80	0.12–194.8	51.334	4.263

The presence of *B. anthracis* (CapB) DNA was significantly associated with elevated levels of organic matter, chromium, cobalt and cadmium while it was significantly associated with low concentrations of magnesium, copper, manganese, sodium, ferrous, calcium and potassium. *F. tularensis* DNA was associated with low concentrations of phosphorous. Except cobalt, the presence of DNA for *C. burnetii* was associated with low concentration of magnesium, sodium, ferrous, calcium and potassium. None of the soil chemistry variables was found to be associated with presence of *B. mallei/pseudomallei* (Table 4).

**Table 4:** **Soil properties and their association with the presence of soil-borne pathogens**.

**Soil component (mg/Kg)**	**Negative**	**Positive**	***F*-test**	***T*-test**
	**Avg. (Range)**	**Avg. (Range)**		
	**(*n* = 131)**	**(*n* = 14)**		
***Bacillus anthracis***				
Organic matter	2.37 (0.0408–6.73)	3.29 (1.25–7.25)	0.357	0.035
Magnesium	11.75 (0.102–82.24)	1.20 (0.33–2.60)	0.000	0.000
Copper	0.42 (0.03–3.083)	0.27 (0.12–0.69)	0.048	0.014
Chromium	0.12 (0.002–0.25)	0.16 (0.12–0.23)	0.003	0.000
Manganese	0.07 (0.02–0.89)	0.029 (0.02–0.04)	0.045	0.000
Cobalt	0.06 (0.004–0.15)	0.092 (0.06–0.13)	0.000	0.001
Cadmium	0.21 (0.03–0.47)	0.27 (0.11–0.42)	0.030	0.024
Sodium	26.85 (0.06–150.70)	8.98 (0.38–15.30)	0.000	0.000
Ferrous	0.63 (0.04–5.59)	0.19 (0.09–0.33)	0.006	0.000
Calcium	23.79 (0.09–103.60)	1.61 (0.12–4.18)	0.000	0.000
Potassium	38.40 (0.12–194.80)	1.01 (0.07–4.70)	0.000	0.000
***Francisella tularensis***				
**Soil component (mg/Kg)**	**(*n* = 126)**	**(*n* = 19)**	***F*****-test**	***T*****-test**
Phosphorus	42.00 (1.00–146.70)	25.55 (5.0–107.20)	0.05	0.013
***Coxiella burnetii***				
**Soil component (mg/Kg)**	**(*n* = 138)**	**(*n* = 07)**	***F*****-test**	***T*****-test**
Magnesium	11.25 (0.12–82.20)	0.56 (0.27–0.68)	0.000	0.000
Cobalt	0.07 (0.005–0.15)	0.10 (0.08–0.14)	0.003	0.002
Sodium	25.05 (0.06–140.70)	7.04 (5.71–8.31)	0.001	0.000
Ferrous	0.61 (0.04–5.59)	0.19 (0.14–0.29)	0.049	0.000
Calcium	22.68 (0.08–103.60)	1.23 (0.98–1.56)	0.000	0.000
Potassium	36.48 (0.12–194.80)	1.53 (0.11–6.53)	0.000	0.000

### Determination of risk factors associated with soil-borne pathogens

Risk factors including presence of domestic animals, distance between the sampling site and animal market, main road and water source, presence of ground cover, animal density and the number of households in the village were evaluated to determine if any of these factors could be associated with the presence or absence of the pathogen at the sampling site (Table 5). It was observed that *B. anthracis* (CapB) and *F. tularensis* were identified in four villages located along the Lahore-Multan road (Figure 1). None of the risk factors were associated with *B. anthracis* (CapB) DNA in the soil sample. The presence of *F. tularensis* in the sample was positively associated with distance from the animal market [2.75 (1.02–7.40)], main road [4.14 (1.23–13.87)] and animal density [2.77 (1.04–7.40)], while for *C. burnetii* in soil sample, it was positively associated with the distance from the main road [32 (5.49–186.3)], vegetation [11.88 (2.17–64.86)] and animal density [5.91 (1.10–31.7)] (Table 5).

**Table 5:** **Risk factors associated with presence or absence of DNA of ***Bacillus anthracis, Francisella tularensis***, and ***Coxiella burnetii*** in soil samples**.

**Criteria**	***Bacillus anthracis***			***Francisella tularensis***			***Coxiella burnetii***		
	**+ve**	**−ve**	**OR (95% CI)**	**+ve**	**−ve**	**OR (95% CI)**	**+ve**	**−ve**	**OR (95% CI)**
**DOMESTIC ANIMAL**									
Present	10	106	0.58 (0.17–2.04)	14	102	0.65 (0.21–2.0)	5	111	0.60 (0.11–3.31)
Absent	4	25		5	24		2	27	
**DISTANCE FROM ANIMAL MARKET**									
>1 km	5	35	1.52 (0.47–4.86)	9	31	2.75 (1.02–7.40)	1	39	0.42 (0.04–3.63)
< 1 km	9	96		10	95		6	99	
**DISTANCE FROM MAIN ROAD**									
>500 m	3	12	2.70 (0.66–11.05)	5	10	4.14 (1.23–13.87)	5	10	32 (5.49–186.3)
< 500 m	11	119		14	116		2	128	
**GROUND COVER**									
Ground cover	4	25	0.58 (0.17–2.04)	5	24	0.65 (0.21–2.01)	5	24	11.88 (2.17–64.86)
No ground cover	10	106		14	102		2	114	
**WATER SOURCE (CANAL/STREAM/DRAIN)**									
< 100 m	10	115	0.34 (0.09–1.24)	15	110	0.54 (0.16–1.85)	2	123	0.048 (0.00–0.27)
>100 m	4	16		4	16		5	15	
**ANIMAL DENSITY**									
< 1000 animals	5	41	1.22 (0.38–3.86)	10	36	2.77 (1.04–7.40)	5	41	5.91 (1.10–31.73)
>1000 animals	9	90		9	90		2	97	
**NO OF HOUSEHOLDS**									
>300 houses/village	10	79	1.64 (0.49–5.53)	8	81	0.40 (0.15–1.07)	6	83	3.98 (0.46–3.93)
< 300 houses/village	4	52		11	45		1	55	

## Discussion

Presumptive diagnosis of a disease condition in humans or animals is largely based on clinical manifestations of the disease for a given geographic area and its population. The process of disease diagnosis is greatly enhanced when there is prior history of a similar disease condition, and this is greatly augmented with accurate clinical, laboratory and epidemiologic data. Therefore, timely identification of the etiologic agent using a validated and approved diagnostic test is critical to developing and implementing the most responsive disease prevention and control practices for a given geographic location and population.

Although in recent years, considerable efforts have been made to improve disease reporting, monitoring and surveillance in Pakistan; much needs to be done with respect to addressing emerging and re-emerging diseases. The Directorate of Animal Disease Reporting and Surveillance (ADRS) and Punjab Health Department in Punjab province of Pakistan periodically collect data on various disease conditions; however, the data collected is largely based upon clinical symptoms and/or with little laboratory diagnostic support. Further reporting for *B. anthracis* and *B. mallei* is exclusively done by ADRS.

In our study, real time PCR was the test of choice for the following reasons: (a) culture-based methods for highly dangerous pathogens such as *B. anthracis, B. mallei/pseudomaillei, C. burnetii, F. tularensis*, and *Y. pestis* requires a highly contained laboratory and trained personnel as these bio-threat pathogens could pose catastrophic human and animal threat in the event there is a lapse in chain of custody and biosafety practices, (b) the RT PCR assays used in the study are validated and are of very high sensitivity and specificity as compared to conventional PCR, and (c) the RT PCR assays allows simultaneous examination of several samples with high throughout and rapid turnaround time. The RT PCR assay and protocol used in our study is highly sensitive (97%) with detection limit as low as < 100 genome copies in a given sample and can be used for a variety of matrices such as tissue, blood, soil and other environmental samples (Christensen et al., 2006). The RT-PCR assay for *C. burnetii* targeted a multicopy gene (IS1111 gene) that has much greater sensitivity than single copy gene targets (Tozer et al., 2014).

The use of manure, plowing during plantation season, or use of waste water and water from small canals used for irrigation of crops/fields could be attributed to prevalence of the soil-borne pathogens. Based on the observations of our study, it is difficult to derive a causal relationship between soil characteristics and the presence/absence of studied pathogens. However, the findings do provide some insights into the distribution of these pathogens in soils of the Lahore region. For instance, pH, organic matter, water activity (a_w_, available water within microenvironment), availability of oxygen, CO_2_/CO_3_ and the presence of certain cations particularly calcium are considered important for the ecology, endemicity and virulence of *B. anthracis* in a given geographical area (Dragon and Rennie, 1995; Shen et al., 2002; Hugh-Jones and Blackburn, 2009; Koehler, 2009; Hammerstrom et al., 2011). While determining the historical distribution and molecular diversity of *B. anthracis* in Kazakhastan, Aikembayev et al. (2010) attributed higher frequency of disease outbreaks in southern and northern portions of the country to alkaline soil rich in organic matter than to central regions which are dominated by desert and where soil does not support the survival of spores. Although we found lower concentration of cations particularly calcium (1.61 mg/Kg or 1610 mEq/gram) at places where DNA to *B. anthracis* was detected than places where it was not detected, it was adequate to support sporulation and its survival in soil. For example, in an effort to determine a possible link between soil calcium and ecology of *B. anthracis*, Smith (personal communication 2003, http://www.oie.int/doc/ged/D7115.PDF, page 12) concluded that areas with calcium (>150 mEq/gram) and pH (>7) had an incidence of disease occurrence seven time more than the places lacking these parameters. Anthrax has been considered endemic in northern Punjab districts such as Chakwal and Jhelum in particular, where sporadic cases do occur during the rainy season. Epp et al. (2010) concluded that within high-risk regions, flooding in spring followed by hot and dry conditions, wet pastures, short grass length and high animal density could result in the persistence and subsequent occurrence of outbreak. Based on our study it can be inferred that soil with alkaline pH and increased organic contents could be suitable for persistence of *B. anthracis*. Virulent strains of *B. anthracis* contain plasmid pXO1 and pXO2 that encode toxins and capsule, respectively (Fouet and Mock, 1996). In our study, DNA of *B. anthracis* that encodes for capsular gene (pXO2, capsular antigen CapB) was identified, while the plasmid for protective antigen (pXO1) was not detected. This could explain the presence of non-virulent type of *B. anthracis* in soil samples from Lahore district and could perhaps explain the lack of any documented evidence of anthrax cases in humans and animals.

The river Ravi which originates from the Himachal Pradesh, India serves as the northwest border of Lahore district (Figure 1). Through recorded history, this fertile river basin has nurtured and supported civilizations and, even in modern times, is fundamental to agriculture, livestock and human habitation. In our study, soil samples from 14 and 19 sample sites were positive for DNA of *B. anthracis* and *F. tularensis*, of which 6 and 7 sampling sites were adjacent to a road way or a canal. Further, both the villages where DNA of *B. mallei/pseudomallei* was identified were exclusively located on “Lahore-Multan road.” This time traveled highway serves as major interstate road that joins the river Ravi at several places of its course. The said road is key to transport people, animals, agricultural and industrial products to other regions of Pakistan. Certain places and villages around the road serve as animal holding areas, auction markets, butcher shops and rest/shelter area for animals and their herders. Many of the positive sample sites were close to private and government-owned slaughter houses as well as animal markets along/around the road. Annually, several thousand animals pass/sheltered for a day or two along the road as well as villages around it. These animals are either sold alive in a nearby animal market or slaughtered for meat. It was also observed that these locations lacked designated areas for waste disposal and animal refuse for slaughter houses in particular. The waste is dispersed into adjacent fields and canals and is being used to irrigate the agriculture land in villages around this interstate road. It is therefore not surprising that we identified pathogens even away from places with more frequent human-animal activity. It was also noted that suburban housing developments and well established industries bordered this road. Based on the findings of the study, it can be inferred that the soil in this area is subject to considerable perturbations through animal, human and industrial activities. The high frequency of detection of all major pathogens in this region of Lahore district is of particular concern to public health and puts human and animal populations at a higher risk of exposure to these pathogens.

None of the soil samples showed the presence of DNA of *Y. pestis*, a vector-borne pathogen transmitted by rat fleas to humans. The long-term persistence of *Y. pestis* in soil and environmental factor contributing its survival is still yet to be fully understood. Perry and Fetherston (1997) reported that *Y. pestis* perishes quickly outside its host or vector, temperature exceeding 40°C and exposure to desiccation. Ayyadurai et al. (2008) showed that *Y. pestis* can be isolated from hydrated soils. In our study, with the exception of May–July, the temperature in the Lahore district is typically below 40°C and the soil remains hydrated through all seasons of the year. Historically, incidence of *Y. pestis* has been reported in coastal borders of sub-continent (India and adjoining areas). However, there has been no reported incidence of plague in the study area, which is consistent with the absence of *Y. pestis* soil DNA reported here.

*F. tularensis* was detected in soil samples from 14 of the 29 villages. As observed with Anthrax, there are no reported incidences or records of disease outbreaks or cases suggestive of tularemia in Lahore district. The incidence of tularemia has been reported globally with varying relative virulence (Low/moderate/high) caused by the *Francisella* species or subspecies involved in the particular geography (Oyston, 2008). A number of cases of clinical infection and subsequent isolation as well as identification of *F. tularensis* has been reported from many countries of the Northern Hemisphere. Further, the strain isolated in North America has been found highly virulent as compared to the strains isolated from Europe or Asia (Sjöstedt, 2007; Oyston, 2008). Though it needs further molecular characterization at subspecies level in future, it is for the first time that DNA to *F. tularensis* has been detected in the environment from this part of the world and thus expanding its known range of occurrence worldwide. It has also been reported that *F. tularensis* may persist in the environment in a given geographical area without concomitant disease outbreaks (Sjöstedt, 2007). Several factors favor the persistence of *F. tularensis* in endemic areas and subsequent infection. These factors include climatic conditions, heat stress, limitation of potassium, cysteine/sulfur, CO_2_ and iron that affects virulence and its survival in the soil (Olsufiev, 1966; Bernard et al., 1994; Deng et al., 2006; Sjöstedt, 2007; Lindgren et al., 2009; Alkhuder et al., 2010). Furthermore, *Francisella* spp. has been found to have affinity to low temperature, moisture, organic matter and hay/straw (Dennis et al., 2001). The role of potential reservoirs cannot be ignored where transmission of virulent strains (*F. tularensis* subspecies *tularensis*) is associated with rabbit, ticks and sheep, whereas, transmission of less virulent strain (*F. tularensis* subspecies *holarctica*) is associated with ponds, streams, lakes, river, and water associated species (Ulu-Kilic and Doganay, 2014). Interestingly, of the total soil sample examined, we found DNA of *F. tularensis* more at places < 100 m of water canals/drains (*n* = 15/19, Table 5).

*Burkhoderia mallei* and *B. pseudomallei* are shown to represent a single genomic species by DNA-DNA hybridization, however each exhibit distinct biochemical properties, epidemiology and manifest different clinical symptoms in humans and animals (Rogul et al., 1970; Coenye and Vandamme, 2003). BLAST analysis was performed for the *B. mallei/pseudomallei* primers and probe used in our study and they were found to be equally applicable to the detection of chromosomal gene of both species. Two soil samples collected along the “Lahore-Multan” road were positive for this pathogen. We anticipated that the likelihood of isolating or identifying this organism was much higher in this region of Lahore compared to other sites as this area has many horses and mule stables and is well traveled for goods/material transport by them. *B. mallei* has been isolated from Pakistan (Hornstra et al., 2009), and asymptomatic horses and mules could transmit this organism without being detected (Hornstra et al., 2009; Khan et al., 2013b). *Burkhoderia mallei*/*pseudomallei* has been shown to survive in the soils and artificial environments involved in animal husbandry such as water troughs (Coenye and Vandamme, 2003; Hornstra et al., 2009) that were observed to be abundant along the roads and in communal stables within our sampling region.

To date, there have been no reported cases/outbreaks of Q-fever in humans and animals in Lahore district. Even in the absence of a reported outbreak, detection of *C. burnetii* in soil (Kersh et al., 2010) or dust and aerosols is not unusual (Schulz et al., 2005; Astobiza et al., 2011). The clinical signs of Q-fever mimic many other diseases with undifferentiated clinical symptoms including fever, nasal and chest congestion, myalgia, neuralgia, nasal and ocular discharges which, in the absence of a confirmed laboratory diagnosis, makes it very difficult to identify the disease (Tozer et al., 2014; Vanderburg et al., 2014).

In conclusion, the findings of our study demonstrate the presence of DNA of *B. anthracis, B. mallei*/*pseudomallei, C. burnetii*, and *F. tularensis* in soil samples collected from Lahore district of Punjab Province. Although we observed an association between the concentration of certain soil analytes and the presence of soil-borne pathogens, a more comprehensive study with a larger sample size will be required to fully examine this observation. Based on the collective experience of the investigators involved in this study, a unified human and animal active and passive surveillance program is key to understand the prevalence and distribution of the pathogens in humans and animals in Pakistan. The surveillance program must be complemented with a contemporary national and region laboratory network system to detect and identify zoonotic diseases of public health importance.

## Author contributions

Conceived and designed the experiment: BMJ, WRM, MZS, MR. Performed the experiment related work in Pakistan: MZS, AAA, TJ, AA, MAA, MABS, MB, AIM, MHC, AB, MN. Sampling and facilitation in relevant procedures: MZS, MAA, MR, KM, KP, SF, TY. Analyzed the data: BMJ, MZS, KP, TJ, AAA, MABS, MB, MHC. Wrote the manuscript: BMJ, MZS.

### Conflict of interest statement

The authors declare that the research was conducted in the absence of any commercial or financial relationships that could be construed as a potential conflict of interest.
